# Effectiveness of an intervention at construction worksites on work engagement, social support, physical workload, and need for recovery: results from a cluster randomized controlled trial

**DOI:** 10.1186/1471-2458-12-1008

**Published:** 2012-11-21

**Authors:** Karen M Oude Hengel, Birgitte M Blatter, Catelijne I Joling, Allard J van der Beek, Paulien M Bongers

**Affiliations:** 1 Netherlands Organisation for Applied Scientific Research TNO, P.O. Box 718, Hoofddorp, AS 2130, The Netherlands; 2 Body@Work, Research Center on Physical Activity, Work and Health, TNO-VU/VUmc, Amsterdam, The Netherlands; 3 365, Utrecht, The Netherlands; 4 Department of Public and Occupational Health, the EMGO Institute for Health and Care Research, VU University Medical Center, Amsterdam, The Netherlands

**Keywords:** Construction industry, Sustainable employability, Intervention study, Empowerment, Physical therapist

## Abstract

**Background:**

To prolong sustainable healthy working lives of construction workers, a worksite prevention program was developed which aimed to improve the health and work ability of construction workers. The aim of the current study was to investigate the effectiveness of this program on social support at work, work engagement, physical workload and need for recovery.

**Methods:**

Fifteen departments from six construction companies participated in this cluster randomized controlled trial; 8 departments (n=171 workers) were randomized to an intervention group and 7 departments (n=122 workers) to a control group. The intervention consisted of two individual training sessions of a physical therapist to lower the physical workload, a Rest-Break tool to improve the balance between work and recovery, and two empowerment training sessions to increase the influence of the construction workers at the worksite. Data on work engagement, social support at work, physical workload, and need for recovery were collected at baseline, and at three, six and 12 months after the start of the intervention using questionnaires.

**Results:**

No differences between the intervention and control group were found for work engagement, social support at work, and need for recovery. At 6 months follow-up, the control group reported a small but statistically significant reduction of physical workload.

**Conclusion:**

The intervention neither improved social support nor work engagement, nor was it effective in reducing the physical workload and need for recovery among construction workers.

**Trial registration:**

NTR1278

## Background

As in many industrialized countries [1], the Dutch construction industry faces the challenges of a decreasing working population. This development can be explained by the fact that less young workers are entering the construction industry and, at the same time, the baby boom cohort moves towards the retirement age [2,3]. To encounter the expected shortages of workers, construction workers need to work longer and retire later than in previous years.

Policies and intervention programs are needed in order to support sustainable employability of construction workers. To develop such interventions, insight in the factors influencing the sustainable employability of construction workers is of interest. Previous studies showed that a poor physical health is an important contributor among blue-collar workers to a diminished ability to continue working until the retirement age [4], and to an earlier retirement [5,6]. Also, among construction workers, mental health was associated with a lower ability to continue working [4]. In addition to health, physically heavy work influences whether workers retire or not. A recent study added that psychosocial factors, such as supervisor support and job autonomy play a significant role in the ability and willingness to continue working as well [4].

Until now, no studies were found on interventions that explicitly aimed to support the sustainable employability among construction workers. Therefore, an intervention program for construction workers was developed using the intervention mapping approach [7], and taking the multi-factorial concept of sustainable employability into account [8]. The prevention program consisted of two individual training sessions of a physical therapist aimed at lowering physical workload, an instrument to raise awareness of the importance of taking rest breaks to reduce fatigue (i.e., Rest-Break tool), and two empowerment training sessions to increase the influence of the construction workers at the worksite [9,10].

In a recent publication, the process of this worksite prevention program was evaluated [11]. The study yielded that the physical therapists and empowerment trainer largely provided the training sessions as intended, but that the Rest-Break tool was poorly implemented. Moreover, the workers and supervisors were moderately satisfied with the program. In addition, the study showed that contextual factors, such as engagement of the top-management, the economic recession, and company size, played an important role during the implementation [11]. Since the effectiveness still has to be established, the aim of the present study was to investigate the effectiveness of the worksite prevention program compared to usual care on social support at work, work engagement, physical workload and need for recovery. In addition, the present study aims to take into account the influence of the process variables and contextual factors on the effectiveness of the intervention.

## Methods

### Study design and study population

The study was a cluster randomized controlled trial (RCT) conducted at the departments of six construction companies, which were specialized in house, commercial or industrial building. Construction workers of these six companies were allowed to participate in the study. Inclusion criteria at baseline were: (i) construction workers were able to complete questionnaires written in the Dutch language, and (ii) construction workers had signed a written informed consent. The study protocol was approved by The Medical Ethics Committee of the VU University Medical Center (Amsterdam, The Netherlands). More details on the study design and methods have been described elsewhere [10].

### Intervention

The intervention was developed using the Intervention Mapping approach, meaning that theoretical information from literature was combined with practical information from stakeholders (employers, supervisors, workers, health professionals, and providers) [7,9]. By applying the Intervention Mapping approach, the intervention is not only tailored to the construction workers but also to the abilities and opportunities of the implementers.

Following from this, a prevention program was developed which consisted of a physical and a mental component. Regarding the physical component, the workers received two individual training sessions of a physical therapist and a Rest-Break tool. During the first training session of the physical therapist, a quick scan questionnaire was followed by a 15-minute observation at the workplace. Based on this, three recommendations on how to reduce the physical workload were written down on a pocket-size card. These recommendations, were for instance, focused on improvements in working style, work methods and rest breaks. Four months later, at the second training session, the experiences so far were discussed and the impact of the advice was evaluated. The second part of the physical component was the introduction of the Rest-Break tool that was constructed by the researchers. This tool aimed to raise awareness about the importance of reducing fatigue by taking flexible rest breaks, and to stimulate to actually take rest breaks. The Rest-Break tool is a flowchart and consists of four steps: (i) the expectations of the workers about their fatigue at the end of the working day, (ii) short-term advice to take mini rest breaks or an additional break of ten minutes, (iii) selection of possible causes of fatigue, and (iv) long-term advice about structurally lowering fatigue. The workers were asked to fill in the tool weekly, alone or with colleagues, and to discuss the results with their supervisor.

As to the mental component, workers received two interactive empowerment training sessions to improve their influence at the worksite. Influence at the worksite could be improved by (i) taking responsibility for their own behaviour and health, (ii) discussing with colleagues about this responsibility, and (iii) improving the communication with the supervisor. The first training session consisted of five steps. During these steps, the workers created a list of topics they wanted to change during the intervention period, and they signed an action plan. Four months later, at the second empowerment training session, the empowerment trainer and workers discussed, evaluated, and reconsidered the action plan as well as the results that were achieved. More details on the development and content of the intervention have been described elsewhere [9].

Workers allocated to the intervention departments received the worksite prevention programme lasting six months, whereas those allocated to the control group received no intervention.

### Randomization, blinding and sample size

Cluster randomization took place at the level of the department within each company, using a computer-generated random-sequence table. In order to avoid intervention group contamination, to accommodate the worksite intervention, and to enhance participants’ compliance, cluster randomization was considered the best randomization strategy for this study. The randomization procedure was performed by a research assistant, who had no prior information about the departments. Obviously, as the intervention took place at the worksite, it was impossible to blind the researchers, the construction workers, their supervisors and the trainers to the allocation. The sample size of workers was calculated according to the number of cases needed to identify an effect on health status which was measured by the SF-12. Health status is one of the other outcome measures of the trial, and will be published in a separate paper. As the SF-12 has rarely been used in intervention studies among the general population, the sample size calculation was based on the SF-36 [12]. Based on means and standard deviations of the SF-36 from earlier studies among different groups of workers, we calculated the sample size needed to detect relevant changes in health, reflecting either “somewhat better (or worse)” or “much better (or worse)” health [12,13]. Because of the cluster randomization design, a certain loss of efficiency associated with cluster randomization relative to individual randomization was taken into account [14]. An effect size of 0.40 was considered to be the lower boundary of a 'medium' effect size [15]. This effect size can be detected with a power (1-β) of 0.80 and a two-tailed alpha of 0.05 with two groups of 100. Taking a loss to follow-up of about 10% into account, 220 workers were required at baseline.

### Outcome measures

For practical reasons, the baseline measurement took place after randomization. Responders on the baseline questionnaire received follow-up questionnaires after three, six and 12 months. The present study investigated the effectiveness of social support at work, work engagement, physical workload, and need for recovery.

#### Social support at work

Social support at work was measured using the Dutch version of the Job Content Questionnaire [16,17]. Co-worker support and supervisory support were measured separately with four items, each on a 4-point rating scale (1=totally disagree; 4=totally agree). These scales have shown moderate to good reliability (Cronbach's alpha between 0.75 and 0.84) [16,17]. A total score of social support at work was obtained by adding the scores of co-worker support to those of supervisory support.

#### Work engagement

Work engagement was measured using a modified version of the Utrecht Work Engagement Scale (UWES-9), which enquires how often the respondents currently experience positive emotions at work [18]. The items were divided into the subscales vigour, dedication, and absorption. In the present study, the items were measured on a 6-point scale ranging from 1 (never) to 6 (always). A total score was obtained by averaging the individual item scores. The psychometric qualities of the UWES-9 have been proven to be acceptable [19].

#### Physical workload

Questions about physical workload were measured using three questions (using force, working in awkward postures and repetitive movements) on a 5-point scale ranging from 1 (never) to 5 (always). These questions were derived from the Periodical Health Screenings survey in the construction industry. This survey is widely used and common among Dutch construction workers, most of whom regularly participate in the Periodical Health Screening. A total score of physical workload was calculated by averaging the three items.

#### Need for recovery

Need for recovery was assessed using an 11-item dichotomized subscale (yes/no) of the VBBA (Dutch questionnaire on Experience and Assessment of Work), which has shown to be valid and reliable (Cronbach's alpha of 0.86) [20,21]. This questionnaire assesses short-term health effects that reflect the worker’s need for recovery at the end of a regular workday [21]. In the present study, the scale was highly skewed to the right, meaning that the majority of the workers reported no fatigue. However, no cut-off point for the scale existed to classify “cases” with high scores on the scale. Based on a previous study on need for recovery [22], the upper quartile of the score in the study was used to define a contrast between workers with considerable need for recovery from work (upper quartile) versus workers with a lower need for recovery from work (lowest three quartiles).

### Statistical analyses

All analyses were performed according to the intention-to-treat principle. Baseline characteristics of the workers in the two groups were compared using the unpaired Student t-test and Pearson’s chi-square test.

To evaluate the effects of the intervention, multilevel analyses were performed for all outcome variables.

Four levels were identified: time (four measurements), worker (n=293), department (n=15), and company (n=6). Linear mixed models were used to evaluate the effects on work engagement, social support and physical workload, and logistic mixed models to evaluate the effects on need for recovery. For each outcome variable, two analyses were performed: 1) crude analysis (i.e. the differences between intervention and control group at three, six and 12 months follow-up, adjusted for corresponding baseline on outcome variable), and 2) adjusted analysis, encompassing the analysis as described above but adjusted for potential confounders. Potential confounders or effect modifiers were measured at baseline (i.e., age, and educational level). Confounding was considered if >10% change occurred in the regression coefficient. Effect modification was considered for age and educational level measured at baseline, using a p-value <0.1 of the interaction term to indicate effect modification. For all analyses the intervention effect of interest was the interaction between group and measurement time. P-values <0.05 were considered to be statistically significant.

To investigate to what extent the implementation influenced the intervention effect, effect modification was also considered on four factors described in the process evaluation. These factors were company size (medium, large), engagement of the top-management towards the program (low, medium, high), year of implementing the program (2008, 2009), and economic recession (company with discharged workers, companies without discharged workers). Furthermore, per-protocol analyses were performed for the number of training sessions that were followed in the intervention group. The linear and logistic regression models were stratified by the number of training sessions followed. The number of training sessions was categorized into three groups; i.e., workers followed none of the training sessions, workers followed one or two training sessions, and workers followed three or four training sessions.

All multilevel statistical analyses were performed using MLwiN version 2.02. All non-multilevel statistical analyses were performed using the Statistical Package of Social Sciences version 17.0 (SPSS Inc, Chicago, IL).

## Results

### Participants

**Figure 1:**
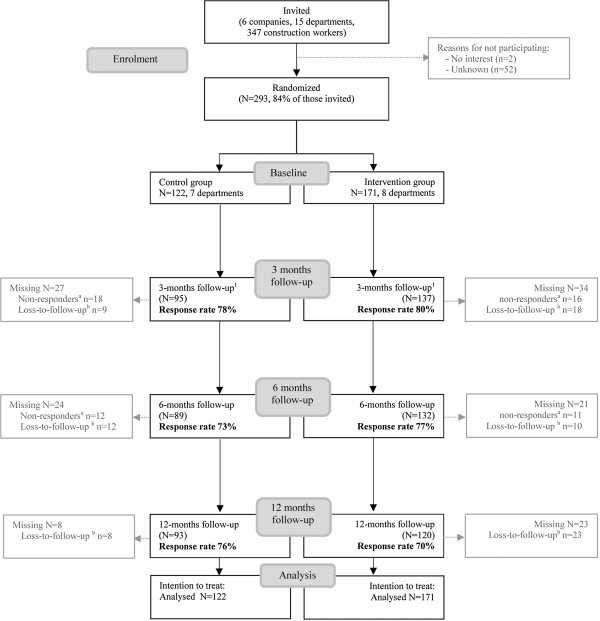
**Flow diagram of the participants through the phases of the trial.**^1^ Workers who were loss-to-follow- up due to non-responding were included again in the following measurements. To illustrate; at three months follow-up, 27 workers did not respond to the questionnaires of whom 18 of them were non-responders. At 6 months questionnaire, 103 (95 +18 workers) workers were approached, and 89 workers responded to  this questionnaire. ^a^ Non-responders was defined as workers that did not complete a particular follow-up measurement. ^b^ Loss-to-follow up was defined as workers that ended participation in follow-up measurements (i.e., drop outs).

Figure 1 outlines the complete flow of the participants from the six companies. Those companies were recruited between March 2008 and December 2009. When a company agreed to participate in the program, construction workers of the company were approached to participate at the worksites, and they received the baseline questionnaire. In total, the baseline questionnaire was distributed to 347 construction workers, of whom 84% (n=293) responded. The randomization procedure allocated 8 departments to the intervention group (n=171) and 7 departments to the control group (n=122). All construction workers were approached for follow-up measurements. Table 1 presents the baseline characteristics of construction workers in the intervention and control group. No significant differences regarding age, gender, profession, and the outcome measures were found between the two groups. However, construction workers in the intervention group were higher educated compared to the construction workers in the control group. After 12 months, the loss-to-follow-up was 24% in the control group and 30% in the intervention group. The main reasons for loss-to-follow-up were that construction workers were on sick leave during the measurements, the contract of construction workers was (un)voluntary ended, and workers were discharged from the company due to the economic crisis. In addition, non-completers were higher educated than completers.

**Table 1:** Baseline characteristics

	**Control group**		**Intervention group**	
	**(n=122)**		**(n=171)**	
**Individual characteristics**				
Age (yr) [mean (SD)]	44.3	(12.7)	41.8	(12.7)
Gender (male) [n (%)]	120	(98%)	171	(100%)
Education [n (%)]*				
Lower education	103	(84%)*	127	(74%)*
Intermediate/higher education	18	(15%)*	44	(26%)*
Missing	1		1	
Profession				
Bricklayer	16	(13%)	39	(23%)
Carpenter	92	(75%)	116	(68%)
Other	14	(12%)	16	(9%)
**Outcomes** [mean (SD)]				
Work engagement (range 1–6) ¥	4.3	(0.8)	4.3	(0.8)
Vigour (range 1–6)	4.3	(0.8)	4.4	(0.8)
Absorption (range 1–6)	4.1	(1.0)	4.0	(1.0)
Dedication (range 1–6)	4.6	(0.9)	4.6	(0.9)
Overall social support (range 8–32) ¥	24.0	(3.4)	24.3	(2.5)
Co-worker support (range 4–16)	12.2	(1.7)	12.4	(1.4)
Supervisor support (range 4–16)	11.8	(2.0)	12.0	(1.7)
Physical workload (range 1–5) ¥	2.6	(0.8)	2.6	(0.8)
Elevated need for recovery [n (%)]	29	(24%)	44	(26%)

Abbreviations: yr, years; SD, standard deviation; n, number; ^*^ p=0.02;

¥ Higher score means a higher level of work engagement (including vigour, absorption and dedication), social support (including co-worker and supervisor support), and physical workload.

### Intervention effects

The means for social support at work, work engagement, physical workload, and need for recovery in the intervention and control group at baseline, and at three, six and 12 months follow-up are shown in Table 2 and Table 3. Additionally, the overall effects of the intervention, and the effects at three, six, and 12 months are presented.

**Table 2:** Intervention effects on social support at work, work engagement, and physical workload between the intervention and control group after three, six and 12 months of follow up

	**Control group**		**Intervention group**			
	**mean**	**(SD)**	**mean**	**(SD)**	**β**	**(95% CI)‡**
**Social support at work**^1^						
*Overall social support (8–32)*						
Baseline	24.0	(3.4)	24.3	(2.5)		
3-months	24.2	(3.1)	24.2	(2.5)	0.02	(−0.61 0.65)
6-months	24.2	(3.2)	25.5	(2.5)	0.25	(−0.40 0.90)
12-months	24.0	(2.9)	23.9	(2.5)	−0.20	(−0.56 0.45)
overall effect					0.03	(−0.39 0.46)
*Co-worker support (range 4–16)*						
Baseline	12.2	(1.7)	12.4	(1.4)		
3-months	12.3	(1.5)	12.3	(1.2)	−0.02	(−0.33 0.30)
6-months	12.3	(1.6)	12.3	(1.4)	0.03	(−0.29 0.35)
12-months	12.2	(1.4)	12.2	(1.3)	−0.02	(−0.35 0.30)
overall effect					0.00	(−0.21 0.20)
*Supervisor support (range 4–16)*						
Baseline	11.8	(2.0)	12.0	(1.7)		
3-months	11.9	(1.9)	11.9	(1.6)	0.07	(−0.34 0.48)
6-months	11.8	(2.0)	12.1	(1.7)	0.27	(−0.15 0.69)
12-months	11.7	(1.8)	11.7	(1.7)	−0.09	(−0.51 0.33)
overall effect					0.09	(−0.18 0.36)
**Work Engagement**^**1**^						
*Work engagement (1–6)*						
Baseline	4.3	(0.8)	4.3	(0.8)		
3-months	4.4	(0.8)	4.2	(0.7)	−0.06	(−0.22 0.11)
6-months	4.2	(0.9)	4.3	(0.8)	0.02	(−0.15 0.19)
12-months	4.2	(0.9)	4.3	(0.8)	0.10	(−0.07 0.27)
overall effect					0.02	(−0.12 0.15)
*Subscale vigour (1–6)*						
Baseline	4.3	(0.8)	4.4	(0.8)		
3-months	4.4	(0.8)	4.3	(0.7)	−0.04	(−0.21 0.13)
6-months	4.2	(0.8)	4.3	(0.8)	0.06	(−0.12 0.24)
12-months	4.3	(0.9)	4.4	(0.8)	0.04	(−0.14 0.22)
overall effect					0.02	(−0.19 0.15)
*Subscale absorption (1–6)*						
Baseline	4.1	(1.0)	4.0	(1.0)		
3-months	4.1	(1.0)	3.8	(1.0)	−0.18	(−0.38 0.02)
6-months	4.1	(1.1)	4.0	(1.0)	−0.07	(−2.00 0.52)
12-months	3.9	(1.1)	4.0	(1.0)	−0.01	(−0.22 0.19)
overall effect					−0.09	(−1.64 1.46)
*Subscale dedication (range 1–6)*						
Baseline	4.6	(0.9)	4.6	(0.9)		
3-months	4.6	(0.9)	4.5	(0.8)	−0.01	(−0.32 0.04)
6-months	4.5	(1.0)	4.5	(0.9)	0.02	(−0.15 0.19)
12-months	4.3	(1.0)	4.5	(0.9)	0.22	(−1.67 2.10)
overall effect					0.07	(−0.08 0.22)
**Physical workload (1–5)**^**2**^						
Baseline	2.6	(0.8)	2.6	(0.8)		
3-months	2.7	(0.8)	2.8	(0.8)	0.09	(−0.08 0.24)
6-months	2.5	(0.7)	2.7	(0.9)	**0.18**	**( 0.01 0.34)**
12-months	2.5	(0.8)	2.6	(0.8)	0.04	(−0.13 0.21)
overall effect					0.10	(−0.02 0.21)

‡ Adjusted model corrected for age and education;

^1^ a positive βèta (β) means a higher engagement (including vigour, absorption and dedication) and social support (including co-worker and supervisor support), and physical workload in the intervention group compared to the control group.

**Table 3:** Intervention effects on need for recovery between the intervention and control group after three, six and 12 months of follow up

	**Control group**	**Intervention group**		
	**%**	**%**	**OR**	**(95% CI)‡**
**Elevated need for recovery**^**1**^				
Baseline	24%	26%		
3-months	26%	31%	1.50	(0.66 3.41)
6-months	25%	26%	1.15	(0.48 2.79)
12-months	27%	26%	0.88	(0.37 2.11)
effect			1.17	(0.66 2.07)

‡ Adjusted model corrected for age and education:

^1^ An odds ratio (OR) above 1 indicates that workers in the intervention group had on average a higher need for recovery compared to the control group.

No significant intervention effects were found for work engagement and the accompanying subscales (i.e. vigour, dedication, and absorption) at three, six and 12 months. Moreover, the intervention did not result in significant effects on social support at work, neither on social support from colleagues nor on social support from the supervisor. Regarding physical workload, a significant intervention effect was found at 6 months follow-up (β 0.18, 95% CI 0.01; 0.34). This effect indicates that construction workers in the intervention group experienced a slightly higher physical workload at 6 months follow-up compared to the construction workers in the control group. Additionally, no overall effect or an effect at any of the time measurements was found for need for recovery. No significant interactions were found for work-related outcomes with age or educational level, indicating that effect modification did not occur.

### Implementation of the intervention

The effect sizes were not influenced by the number of followed training session of the workers in the intervention group. Moreover, the effectiveness of the intervention on the outcomes did generally not differ between medium and large companies, between companies with a low, medium, and high engagement of the top-management towards the program, between companies with and without discharged workers, and between companies that started the intervention in 2008 compared to those that started the intervention on 2009.

## Discussion

The present study showed that the prevention program among construction workers was not effective in improving social support at work and work engagement, nor in reducing physical workload and need for recovery. At 6 months follow-up, the control group reported a small but statistically significant reduction of physical workload.

This study is the first prospective controlled trial aimed to support sustainable employability in the construction industry by means of an intervention consisting of a physical component and mental component. A balance between good health and work was suggested as important to support sustainable employability during the development of the program [9], as well as by previous studies [4-6]. Until now, most health promotion programs in the construction industry have focused on either improving the health of construction workers by means of a lifestyle program [23,24], or on decreasing the work demands by means of ergonomic measures [25].

Both the intervention and control group did not show any significant differences for social support at work and work engagement. Despite the fact that psychosocial factors have been recognized as factors associated with musculoskeletal symptoms [26,27] and short-term sickness absence [27], intervention studies among blue-collar workers did not focus on the psychosocial aspects of work yet. Regarding physical workload, the present study showed no overall intervention effect. However, the intervention group reported a significant higher physical workload at 6 months follow-up. It should be noticed that this adverse effect in absolute numbers was very small. A previous review recommended that an education program or involvement of workers combined with ergonomic measures might be more promising to reduce workload [28]. Also, no intervention effect was found on decreasing the elevated need for recovery among the construction workers.

Strengths of the study include the cluster RCT design, and the high participation rate among the workers. Although participation of blue-collar workers in intervention studies is usually low [29], 84% of the construction workers approached in the present study were willing to participate in the intervention. These strengths improve the generalizability of the study findings towards workers in the construction industry. Randomization at department level is another strength that minimized possible contamination between the construction workers from the intervention group and control group. Avoiding contamination is especially important in this industry where workers are working at worksites that are temporary and mobile.

Some methodological considerations deserve attention as well. First, the study design was two-armed (control versus intervention), which does not allow a separate evaluation of the individual components of the prevention program. As a consequence, the (in-)effectiveness of the program can only be attributed to the entire program. Second, the sample size calculation was based on a change in health status. The sample size might therefore be too small to detect a significant change in outcomes measures. To illustrate, another study calculated that almost 250 workers were needed in each group to find an effect on work engagement [30]. However, while providing sufficient statistical power would have diminished the confidence intervals, these smaller confidence intervals would still not have led to statistically significant intervention effects as the mean scores between the intervention and control group are quite similar for most outcomes (Table 2 and Table 3). Third, data were obtained solely from questionnaires. As a result, all data were self-reported, inducing a potential risk of bias due to socially desirable answers. Fourth, participation in the program was voluntary, and bias due to non-response could therefore not be ruled out in intervention studies. However, the participation of workers was very high (84%), indicating that selection bias due to non-response was minimal in the current study. Fifth, the loss-to-follow-up was higher than expected due to the economic crisis and health-related absenteeism of the workers. As a consequence of the economic recession, one company was forced to lay-off workers, and to offer the remaining workers a temporary part-time job during the intervention program. Participants who were lost-to-follow up were higher educated. However, as no other differences between completers and non-completers were found, we assume the bias due to selective loss-to-follow-up was limited.

Based on the results obtained from the present RCT, it can be concluded that no intervention effects on any of the outcomes were found. Possible reasons for ineffectiveness can be distinguished into program failure and theory failure [31]. Program failure indicates that a poorly implemented intervention resulted in no improvement on the study outcomes. Theory failure implicates that an intervention has been perfectly implemented, but did not lead to improvement on the study outcomes. Both types of failure have taken place in the present study.

Some clear signs of program failure were detected in the intervention. First, the effectiveness might be dimmed by the moderate compliance. Although the training sessions were incorporated into the existing health and safety program of the Dutch construction industry, 39% of the construction workers followed less than three training sessions. In addition, construction workers who followed the training sessions of the physical therapist were satisfied about the personal contact and individual advices. However, their opinion about the training session of the empowerment trainer varied. This might be explained by the fact that the empowerment training sessions aimed to change work on an organization level, which was new for both supervisors and workers. The moderate compliance and lower satisfaction towards the training session of the empowerment trainer might have dimmed the effectiveness of the intervention. However, the per-protocol analyses on the number of training sessions showed no differences between workers with low or high compliance. Additionally, the Rest Break Tool was filled in by less than half of the construction workers [11]. Therefore, it is plausible that outcomes closely related to the Rest-Break tool such as need for recovery showed no differences between the intervention and control group. Second, the intervention could be less effective because the rationale behind the intervention was not perfectly implemented by the trainers [11]. For instance, the physical therapist did not deliver all training sessions individually, and the empowerment trainer did not always involve the supervisor in the training sessions. Third, the intervention could be less effective due to the economic climate. During the worldwide crisis, companies and their workers might feel obliged to only focus on activities that are obviously and directly contributing to the productivity at the worksites, and not on prevention programs. Moreover, construction workers may not have entirely committed themselves to the prevention program if they face the fear of losing their jobs at the same time [32].

Although the lack of effect can be caused by program failure, the question arises whether the intervention would be effective if the compliance was optimal, and all trainers delivered the training as intended. Because no improvements on the outcomes were detected at all, it is plausible that the rationale behind the intervention is not entirely correct. First, construction workers showed low interest in the application of this tool as they experienced difficulties filling in their weekly status of fatigue, and they mentioned that the advice was not always feasible in daily practices. As frequency and duration of rest breaks are recorded in policies at worksite or company level, involvement of supervisors and middle-management is essential to take additional rest breaks, and consequently reduce fatigue. Second. the construction workers in the present study mentioned that involvement of the supervisors and management could be valuable in the empowerment training sessions as well [11]. In addition to the rest breaks, achieving a change in topics such as more communication at the worksite, and asking for assistance lies not only within the power of the workers, but relies also on the decision of supervisor and middle-management (e.g., organizational level). Therefore, a more shared responsibility between construction workers, supervisors and middle-management is needed to integrate social support, work engagement and rest breaks more deeply in the work culture of the companies. Although the supervisors were invited to attend the empowerment training sessions, they were mostly not attending these meetings. In addition to the involvement of supervisors and middle-management, a change in topics as described in the empowerment sessions was also difficult to achieve at the worksite because of the economic recession. For instance, workers might have been hindered to take additional rest breaks at a time when job security was threatened by the economic recession, whereas supervisors might have been less willing to accept the additional rest breaks during these times.

## Conclusion

In conclusion, the prevention program was not effective with regard to work engagement, social support at work, physical jobs demands, and need for recovery. Moreover, the effectiveness of the intervention was not influenced by number of training sessions followed, company size, economic recession, engagement of the top-management towards the program, and intervention year.

## Competing interest

The authors declare that they have no competing interests.

## Authors’ contribution

All authors contributed to the design of the study. CJ wrote the initial research proposal. KOH is the principle researcher, and was responsible for the data collection and analyses. BB, AvdB, and PB supervised the study. All authors approved the final manuscript.

## Pre-publication history

The pre-publication history for this paper can be accessed here:

http://www.biomedcentral.com/1471-2458/12/1008/prepub

## References

[B1] Eurostat Europe in Figures; Eurostat yearbook 2011, chapter 5 labour market 2011 Luxembourg: Eurostat European Commission

[B2] Beereboom HJA Blomsma G Corten IW Muchall S De bouwarbeidsmarkt in de periode 2005–2010 [The labour market of the construction industry in the period 2005–2010] 2005 Amsterdam: Economisch Insituut voor de bouw

[B3] Sijpersma R De oudere werknemer in de bouw. [The older worker in the construction industry] 2003 Amsterdam: Economisch Insituut voor de bouw

[B4] Oude Hengel KM Blatter BM Geuskens GA Koppes LLJ Bongers PM Factors associated with the ability and willingness to continue working until the age of 65 in construction workers Int Arch Occup Environ Health 2011 85 7 783 90 22109674 10.1007/s00420-011-0719-3

[B5] Szubert Z Sobala W Current determinants of early retirement among blue collar workers in Poland Int J Occup Med Environ Health 2005 18 2 177 184 16201209

[B6] Lund T Iversen L Poulsen KB Work environment factors, health, lifestyle and marital status as predictors of job change and early retirement in physically heavy occupations Am J Ind Med 2001 40 2 161 169 10.1002/ajim.1084 11494344

[B7] Bartholomew LK Parcel GS Kok G Gottlieb NH Planning health programs: an intervention mapping approach 2006 San Francisco: Jossey-Bass

[B8] Shacklock K Brunetto Y A model of older workers' intentions to continue working Pers Rev 2011 40 2 252 274 10.1108/00483481111106110

[B9] Oude Hengel KM Joling CI Proper KI Van der Molen HF Bongers PM Using intervention mapping to develop a worksite prevention program for construction workers Am J Health Promot 2010 26 e1 e10 21879927 10.4278/ajhp.100326-QUAL-88

[B10] Oude Hengel KM Joling CI Proper KI Blatter BM Bongers PM A worksite prevention program for construction workers: design of a randomized controlled trial BMC Publ Health 2010 10 10.1186/1471-2458-10-336 PMC2896359 20546568

[B11] Oude Hengel KM Blatter BM Van der Molen HF Joling CI Proper KI Bongers PM Van der Beek AJ Meeting the challenges of implementing an intervention to promote work ability and health-related quality of life at construction worksites: a process evaluation J Occup Environ Med 2011 53 12 1483 1491 10.1097/JOM.0b013e3182398e03 22104978

[B12] Aaronson NK Muller M Cohen PD Essink-Bot ML Fekkes M Sanderman R Sprangers MA Te Velde A Verrips E Translation, validation, and norming of the Dutch language version of the SF-36 Health Survey in community and chronic disease populations J Clin Epidemiol 1998 51 11 1055 1068 10.1016/S0895-4356(98)00097-3 9817123

[B13] Beaton DE Hogg-Johnson S Bombardier C Evaluating changes in health status: reliability and responsiveness of five generic health status measures in workers with musculoskeletal disorders J Clin Epidemiol 1997 50 1 79 93 10.1016/S0895-4356(96)00296-X 9048693

[B14] Simpson JM Klar N Donnor A Accounting for cluster randomization: a review of primary prevention trials, 1990 through 1993 Am J Public Health 1995 85 10 1378 1383 10.2105/AJPH.85.10.1378 7573621 PMC1615612

[B15] Cohen J Statistical Power Analysis for the Behavioral Sciences 1988 New York: Lawrence Erlbaum Associates

[B16] Karasek R Brisson C Kawakami N Houtman I Bongers P Amick B The Job Content Questionnaire (JCQ): an instrument for internationally comparative assessments of psychosocial job characteristics J Occup Health Psychol 1998 3 4 322 355 9805280 10.1037//1076-8998.3.4.322

[B17] Karasek RA Job content questionnaire and user's guide 1985 Lowel: University of Massachusetts Lowell, Department of Work Environment

[B18] Schaufeli WB Bakker AB Salanova M The measurement of work engagement with a short questionnaire: a cross-national study Educ Psychol Meas 2006 66 4 701 716 10.1177/0013164405282471

[B19] Schaufeli W Bakker A Utrecht work engagement scale 2003 Utecht: Occupational Health Psychology Unit

[B20] Van Veldhoven M Broersen S Measurement quality and validity of the " need for recovery scale" Occup Environ Med 2003 60 i3 i9 10.1136/oem.60.suppl_1.i3 12782740 PMC1765728

[B21] Van Veldhoven M Meijman TF Broersen JPJ Fortuin RJ Handleiding VBBA [User's guide VBBA] 2002 Amsterdam: SKB Vragenlijst Services

[B22] Van Amelsvoort LG Jansen NW Swaen GM Van den Brandt PA Kant I Direction of shift rotation among three-shift workers in relation to psychological health and work-family conflict Scand J Work Environ Health 2004 30 2 149 156 10.5271/sjweh.772 15143742

[B23] Groeneveld IF Proper KI Van der Beek AJ Van Mechelen W Sustained body weight reduction by an individual-based lifestyle intervention for workers in the construction industry at risk for cardiovascular disease: results of a randomized controlled trial Prev Med 2010 51 3–4 240 246 20692282 10.1016/j.ypmed.2010.07.021

[B24] Ludewig PM Borstad JD Effects of a home exercise programme on shoulder pain and functional status in construction workers Occup Environ Med 2003 60 11 841 849 10.1136/oem.60.11.841 14573714 PMC1740414

[B25] Luijsterburg PA Bongers PM de Vroome EM A new bricklayers' method for use in the construction industry Scand J Work Environ Health 2005 31 5 394 400 10.5271/sjweh.923 16273966

[B26] IJzelenberg W Molenaar D Burdorf A Different risk factors for musculoskeletal complaints and musculoskeletal sickness absence Scand J Work Environ Health 2004 30 1 56 63 10.5271/sjweh.765 15018029

[B27] Morken T Riise T Moen B Hauge SH Holien S Langedrag A Pedersen S Saue IL Seljebo GM Thoppil V Low back pain and widespread pain predict sickness absence among industrial workers BMC Musculoskelet Disord 2003 4 21 10.1186/1471-2474-4-21 12956891 PMC200978

[B28] Van der Molen HF Sluiter JK Hulshof CT Vink P Frings-Dresen MH Effectiveness of measures and implementation strategies in reducing physical work demands due to manual handling at work Scand J Work Environ Health 2005 31 Suppl 2 75 87 16363450

[B29] Glasgow RE McCaul KD Fisher KJ Participation in worksite health promotion: a critique of the literature and recommendations for future practice Health Educ Q 1993 20 3 391 408 10.1177/109019819302000309 8307762

[B30] Van Berkel J Proper KI Boot CR Bongers PM Van der Beek AJ Mindful "Vitality in Practice": an intervention to improve the work engagement and energy balance among workers; the development and design of the randomised controlled trial BMC Publ Health 2011 11 10.1186/1471-2458-11-736 PMC3189893 21951433

[B31] Kristensen TS Intervention studies in occupational epidemiology Occup Environ Med 2005 62 3 205 210 10.1136/oem.2004.016097 15723887 PMC1740975

[B32] Hodgins M Battel-Kirk B Asgeirsdottir AG Building capacity in workplace health promotion: the case of the healthy together e-learning project Glob Health Promot 2010 17 1 60 68 10.1177/1757975909356629 20357353

